# Effects of Fentanyl on Emergence Agitation in Children under Sevoflurane Anesthesia: Meta-Analysis of Randomized Controlled Trials

**DOI:** 10.1371/journal.pone.0135244

**Published:** 2015-08-14

**Authors:** Fenmei Shi, Ying Xiao, Wei Xiong, Qin Zhou, Peng Yang, Xiongqing Huang

**Affiliations:** 1 Department of Anaesthesiology, Sun Yat-sen Memorial Hospital, Sun Yat-sen University, Guangzhou, China; 2 Department of Anaesthesiology, The First Affiliated Hospital, Sun Yat-sen University, Guangzhou, China; Bambino Gesù Children's Hospital, ITALY

## Abstract

**Background and Objectives:**

The goal of this meta-analysis study was to assess the effects of fentanyl on emergence agitation (EA) under sevoflurane anesthesia in children.

**Subjects and Methods:**

We searched electronic databases (PubMed, Embase, Web of Science and the Cochrane Central Register of Controlled Trials) for articles published until December 2014. Randomized controlled trials (RCTs) that assessed the effects of fentanyl and placebo on EA under sevoflurane anesthesia in children that the outcome were the incidence of EA, postoperative pain, emergence time or adverse effects were included in this meta-analysis.

**Results:**

A total of 16 studies, including 1362 patients (737 patients for the fentanyl group and 625 for the placebo group), were evaluated in final analysis. We found that administration of fentanyl decreased the incidences of EA (RR = 0.37, 95% CI 0.27~0.49, *P*<0.00001) and postoperative pain (RR = 0.59, 95% CI 0.41~0.85, *P* = 0.004) but increased the incidence of postoperative nausea and vomiting (PONV) (RR = 2.23, 95% CI 1.33~3.77, *P* = 0.003). The extubation time (WMD = 0.71 min, 95% CI 0.12~1.3, P = 0.02), emergence time (WMD = 4.90 min, 95% CI 2.49~7.30, *P*<0.0001), and time in the postanesthesia care unit (PACU) (WMD = 2.65 min, 95% CI 0.76~4.53, *P* = 0.006) were slightly increased. There were no significant differences in the time to discharge of day patients (WMD = 3.72 min, 95% CI -2.80~10.24, *P* = 0.26).

**Conclusion:**

Our meta-analysis suggests that fentanyl decreases the incidence of EA under sevoflurane anesthesia in children and postoperative pain, but has a higher incidence of PONV. Considering the inherent limitations of the included studies, more RCTs with extensive follow-up should be performed to validate our findings in the future.

## Introduction

Emergence agitation (EA), is common that occurs during the early stage of recovery from general anesthesia in children, particularly in those under sevoflurane anesthesia [1]. Behavioral changes after general anesthesia in children have been described using different descriptive terms in different studies, such as ‘agitation’, ‘excitation’ and ‘delirium’. The definition of this condition has been described as ‘a mental disturbance during recovery from general anaesthesia that may consist of hallucinations, delusions and confusion manifested by moaning, restlessness, involuntary physical activity and thrashing about in the bed [2]. Emergence delirium is an extreme form of EA which is described as ‘a disturbance in a child’s awareness of and attention to his/her environment with disorientation and perceptual alterations’ and not all agitated children are truly delirious [3, 4]. We use the term ‘emergence agitation’ to encompass this clinical entity for the purpose of this meta-analysis.

EA was first described in the early 1960s [3]. Depending on the definition and evaluation methods adopted, the prevalence of EA is between 2% and 80%[5], and it is more common in preschool children. EA is attributed to many factors, such as age, rapid awakening after surgery, pain, anxiety before anesthesia, type of surgery, individuality of children, and anesthetics used. Pain and EA can overlap and it is difficult to distinguish the two phenomenon [6]. Although EA is generally self-limited, it can be severe and may result in physical harm to the child, the need for further post-anesthesia care and eventually supplemental sedative or analgesic drugs [7, 8]. Also, an unsettle behaviour reduces parental and caregivers’ satisfaction. Long-term psychological implications of early postoperative negative behavior are still unclear, but the new-onset postoperative maladaptive behavioral changes including separation anxiety, apathy and withdrawal, eating problems, and sleep problems are closely associated with EA [9].

Different strategies have been suggested for decreasing the incidence and severity of EA, such as the administration of sedative medication before induction and changes in the anesthesia maintenance technique [5, 10, 11]. Drugs such as fentanyl may reduce the incidence of EA under sevoflurane anesthesia. Fentanyl is a potent opioid receptor agonist with sedative and analgesic effects. It is routinely used in the practice of pediatric perioperative medicine. Some clinical trials have shown that fentanyl can prevent EA under sevoflurane anesthesia in children [12, 13]. However, no meta-analysis based on the available randomized trials in the literature has been conducted. Therefore, we conducted a systematic review to compare the effect of fentanyl and placebo on emergence agitation in children under sevoflurane anesthesia.

## Methods

The prospective protocol, literature searching strategies, inclusion and exclusion criteria, outcome measurements, and statistical analysis methods used were based on the recommendations of the PRISMA statement and the Cochrane Collaboration for systematic reviews and meta-analysis [14, 15].

### Literature search strategy

A comprehensive literature search was performed in December 2014. We searched electronic databases, including PubMed, Embase, Web of Science, and the Cochrane Central Register of Controlled Trial. The key search terms were as follows: sevofluran*, emergence agitation/ excit*/ delirium/ confusion, (postoperative/ postanesthetic) (agitation/ confusion / behavioral change*), children/ infant, and fentanyl. The manual searching of the references of the retrieved studies were used to extend the search. Only English articles were considered. When necessary, we contacted the authors for additional unpublished data.

### Inclusion and exclusion criteria

RCTs comparing fentanyl with placebo (normal saline) administered perioperatively to reduce EA incidence in pediatric patients (aged 1–14 years) with sevoflurane anesthesia were included in this systematic review. We excluded letters to the editor, editorials, case reports, reviews, and animal studies.

### Data extraction and outcome measurements

Two independent authors extracted and summarized data from eligible trials. Disagreements were resolved by discussion with other authors. We extracted the following data from each eligible trial: first author, publication year, patient ages, type of surgery, number of patients, sedative premedication, dose, timing, and route of administration of fentanyl/placebo, sevoflurane anesthesia protocol, perioperative analgesia, the EA incidence, postoperative pain, emergence time, extubation time, time in postanesthesia care unit (PACU), time to discharge and adverse events.

The primary outcome is the incidence of emergence agitation (EA). EA incidence was defined as the incidence of participants with postoperative behavioural disturbance during emergence from anesthesia, which was measured by the authors of included studies. The secondary outcomes examined in this study included pain incidence in PACU, extubation time, emergence time, time in the PACU, the time to discharge of day patients and adverse events, such as the incidence of PONV, respiratory adverse events and haemodynamic changes requiring intervention. Pain incidence in PACU was defined by the authors of the studies using the Objective Pain Scale (OPS), Children’s and Infant’s Postoperative Pain Scale (CHIPPS) or four-point Verbal Rating Scale. Extubation time was defined as the time interval from anesthetic discontinuation to extubation. Emergence time was measured as the time between discontinuation of anesthesia and spontaneous eye opening. Time in the PACU was defined as the time interval from anesthetic discontinuation to discharge from the PACU. The time to discharge of day patients was defined as the time between anesthetic discontinuation and discharge from the hospital of day patients. The incidence of PONV was assessed by evaluating nausea and vomiting behaviors from the entrance of patients into the PACU to 24 h after surgery.

### Quality assessment and statistical analysis

We examined the quality of studies included in the meta-analysis using the Cochrane Collaboration’s tool for assessing risk of bias [16]. The domains included a random sequence generation, allocation concealment, blinding of participants and personnel, blinding of outcome assessment, incomplete outcome data and selective reporting.

Meta-analyses were conducted using Review Manager 5.3 (Cochrane Collaboration, Oxford, UK) and Stata software, version 12.0 (Stata Corporation, College Station, TX). Relative risks (RRs) and weighted mean differences (WMDs) were used to compare dichotomous and continuous variables, respectively, both with corresponding 95% confidence intervals (CIs). A confidence interval for an RR of <1 indicated that the incidence of the test target in the fentanyl group was lower than that in the placebo group. If studies presented continuous data as median and range values, the means and standard deviations were transformed as described by Hozo *et al*.[17]

Statistical heterogeneity was assessed using the chi-square test with a significance of *P*<0.10 [18]. Heterogeneity was quantified with the *I* ^*2*^ statistic [19]. If *P*>0.10 and *I* ^*2*^<50%, fixed effects analysis was conducted to calculate the pooled OR; otherwise, a random effects model was used [16]. We conducted subgroup analyses to investigate possible causes of heterogeneity. Sensitivity analyses were performed by removing each trial individually to evaluate the quality and consistency of the results. To evaluate whether potential publication bias might have affected statistical results, we applied funnel plots, Begg’s test and Egger’s test. All statistical tests were 2-sided.

## Results

### Evidence synthesis

**Fig 1:**
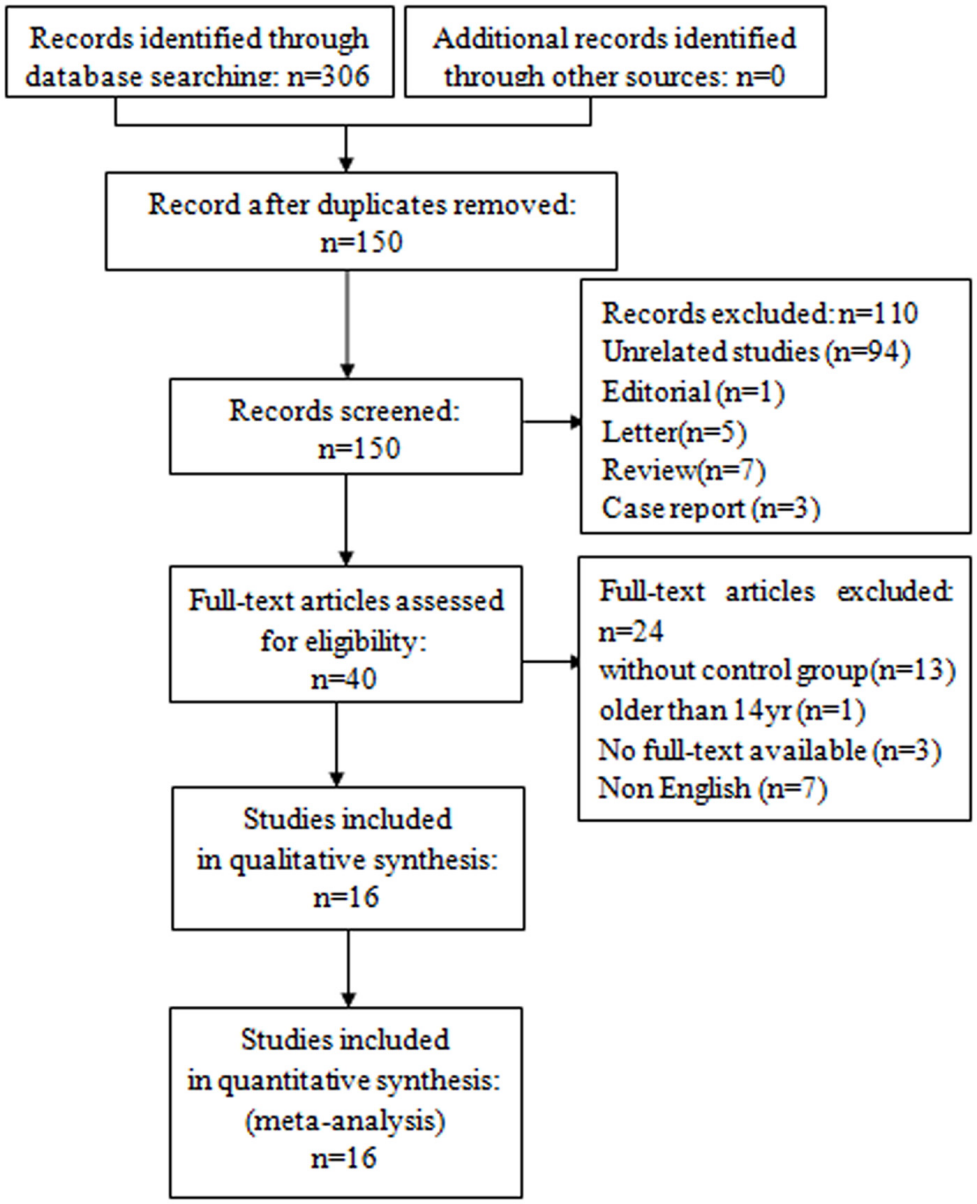
Flow diagram of studies identified, included, and excluded.

Our initial search yielded 306 studies. After removing 156 duplicate studies, we evaluated the abstracts of 150 studies. From this evaluation, 94 studies were excluded as unrelated, 1 was excluded as editorial, 5 were excluded as letters, 7 were excluded as reviews, and 3 were excluded as case reports. The full-text review of 40 studies led to the exclusion of 24 for the following reasons: 13 for the lack of a control group [20–31], 1 for being older than 14 years [32], 3 for the lack of availability of a full-text version [33–35] and 7 for not being written in English [36–42]. Therefore, 16 studies [4, 12, 43–56], including 1362 cases (737 cases for the fentanyl group and 625 cases for the placebo group), reached the predefined inclusion criteria and were finally included in our analysis (Fig 1).

### Characteristics of eligible studies

The characteristics of the included studies are shown in Table 1. Among these studies, two different fentanyl doses were introduced in 3 trials [12, 48, 53]. For the trials that compared a control group with multiple intervention groups using different fentanyl doses, we combined the intervention groups to conduct a single pair-wise comparison. Single fentanyl administration was performed in 15 trials [4, 12, 43–47, 49–56], and continuous infusion was carried out in 1 trial [48]. Five studies were performed in the USA [12, 46, 53–55], three in Egypt [43, 47, 56], two each in Korea [44, 49] and Turkey [50, 52], and one each in Italy [4], China [45], Japan [48] and Saudi Arabia [51].

**Table 1:** Characteristicsof included studies.

Author year	Age	Surgery	Study/control	Study intervention	Premedication	Analgesics	Regional block	Assessment methods of EA
**Borton 2014**	2-11yr	subumbilical surgery	29/29	Fentanyl 2ug/kg iv before surgery	midazolam 0.5mg/kg(Oral)	Acetaminophen 40mg/kg	Iilio-inguinal/iliohypogastric block or Penileblock or caudal block	ED:PAED ≥ 12. EA:Cravero score≥4
**Rashad 2014**	1–3 yr	Ambulatory hypospadias repair	20/20	Fentanyl 1ug/kg iv before the end of surgery	No	No	Caudal block	Cravero Scale≥4
**Kim 2013**	1.5–6 yr	Ambulatoryinguinal hernia repair	66/70	Fentanyl 1ug/kg iv before the end of surgery	No	No	Caudal block	Aono’s scale≥3, or Cravero scale≥4
**Li2011**	3-11yr	Adenotonsillectomy	34/34	Fentanyl 2ug/kg iv after induction	No	Tramadol 2 mg/kg and dexamethasone0.1 mg/kg	No	Aono’s scale≥3
**Pestieau 2011**	0.5-6yr	BMT	23/27	Fentanyl 2ug/kg intranasal after induction	No	No	No	Watcha scale≥2
**Asaad 2011**	5-10yr	Inguinal hernia repair, hydrocele, or circumcision	28/30	Fentanyl 1ug/kg iv after intubation	No	No	Caudal block	Aono’s scale≥3
**Inomata 2010**	2-6yr	Minor surface surgery	93/46	Fentanyl1ug/kg (2ug/kg) iv and continuous infusion 0.5ug/kg/h(1ug/kg/h) before intubation	No	No	Field block	PAED>10
**Jung 2010**	3–10 yr	Stabismusor entropion surgery	49/44	Fentanyl 1.5ug/kg iv after induction	No	Ketorolac 0.5mg/kgOndansetron0.1 mg/kg	No	Cohen scale = 3
**Erdil 2009**	2-7yr	adenoidectomy with or without BMT	30/30	Fentanyl2.5ug/kg iv after induction	Paracetamol 40mg/kg (rectally)	Dexamethasone 0.5mg/kg	No	5-point scale≥4
**Makharita 2009**	3-8yr	BMT	40/40	Fentanyl 1ug/kg iv before the end of surgery	Acetaminophen 40mg/kg (rectally)	No	No	Aono’s≥3
**Bakhamees2009**	2-6yr	adenotonsillectomy with or without BMT	40/40	Fentanyl 1.5ug/kg iv after intubation	midazolam 0.5mg/kg(Oral)	Paracetamol 40mg/kg rectal	No	10-point scale≥2
**Demirbilek2004**	2-7yr	Adenoidectomy or tonsillectomy or both	30/30	Fentanyl 2.5ug/kg iv after induction	Midazolam 0.5mg/kg orally	Acetaminophen 30mg/kg rectal	No	Cohen scale = 3
**Binstock2004**	2-10yr	Outpatient procedure	74/51	OTFC10-15ug/kg (100ug)before induction	OFTC10-15ug/kg(100ug/kg)vs. No	Bupivacaine0.125%,1ml/kgcaudal block	Bupivacaine 0.125%, 1ml/kg Caudal block	Anxiety/agitation≥2
**Cravero 2003**	1.5-10yr	MRI scanning	16/16	Fentanyl 1ug/kg iv before end of surgery	No	No	No	Cravero scale≥4
**Finkel 2001**	0.5-5yr	BMT	101/49	Fentanyl 1ug/kg(2ug/kg) intranasal after induction	No	Acetaminophen 40mg/kg (rectally)	No	Watcha scale≥3
**Galinkin 2000**	0.75–6 yr	BMT	64/69	Fentanyl 2ug/kg intranasal after induction	Acetaminophen10mg/kg, midazolam 0.5 mg/kg orally	No	No	Aono’s scale≥3

BMT = bilateral myringotomy and tubes, MRI = magnetic resonance imaging, ND = not determined, PACU = post anesthesia care unit; Oral transmucosal fentanyl citrate = OTFC

### Primary outcomes

#### EA incidence

**Fig 2:**
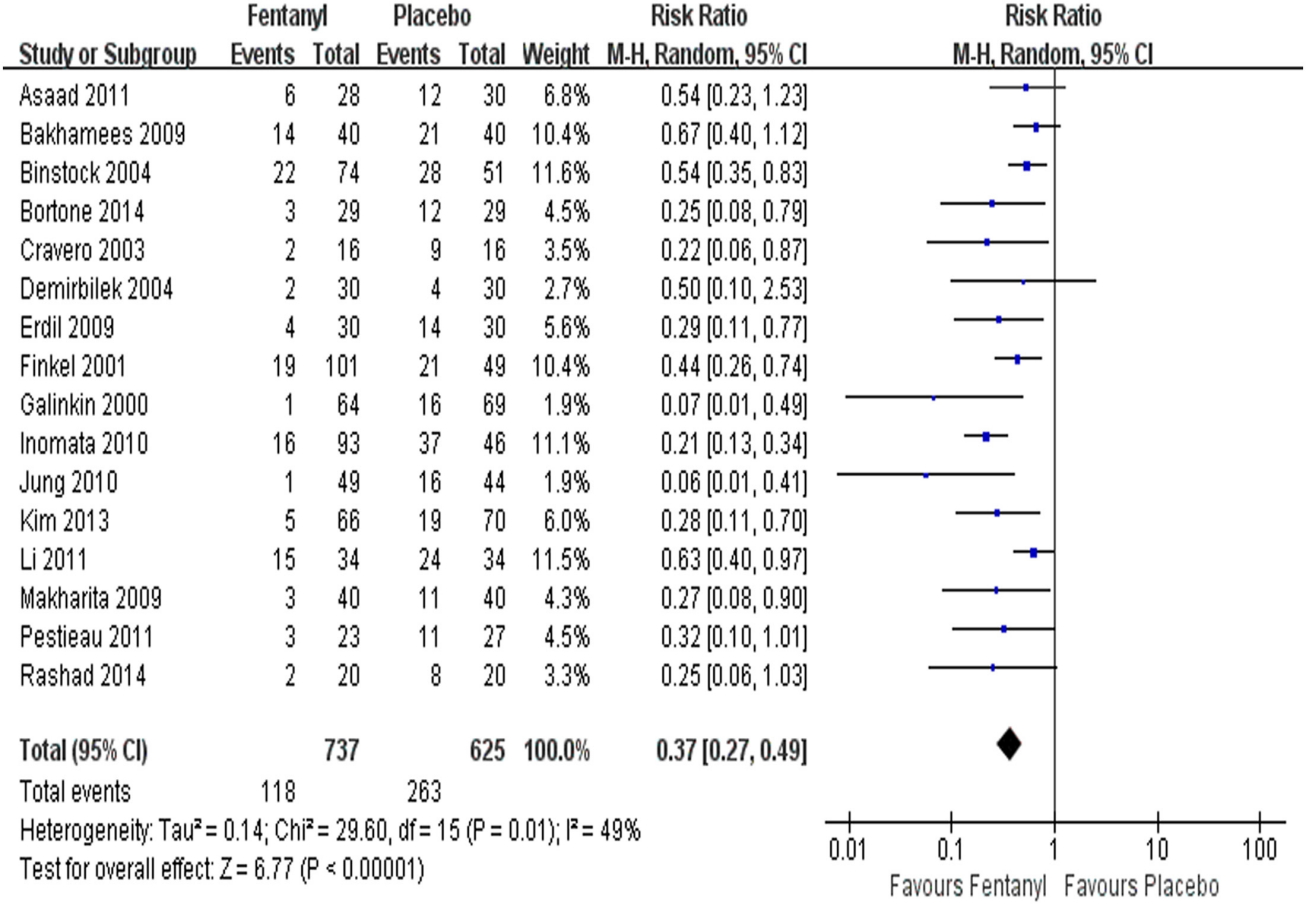
Forest plot and meta-analysis of EA incidence. EA = emergence agitation; M-H = Mantel-Haenszel method; CI = confidence interval.

Sixteen studies [4, 12, 43–56] (n = 1362) reported the incidence of EA and were included in pooled analysis of fentanyl vs. placebo (Fig 2). There was strong evidence that fentanyl significantly reduced the incidence of EA in children with sevoflurane anesthesia(RR = 0.37, 95% CI 0.27~0.49, *P*<0.00001, *I* ^2^ = 49%) (Table 2).

**Table 2:** Meta-analysis results of all items.

Items	No. of studies	No. of participants	Effect size (95%CI)	*P*-value	*I* ^*2*^,%	Heterogeneity *P*-value
**EA**	16	1362	RR 0.37(0.27,0.49)	<0.00001	49	0.01
**Pain**	5	308	RR 0.59(0.41,0.85)	0.004	0	0.65
**Extubation time, min**	5	420	WMD 0.71(0.12,1.3)	0.02	0	0.79
**Emergence time, min**	8	587	WMD 4.9(2.49,7.3)	<0.0001	95	<0.00001
**Time of PACU, min**	13	1175	WMD 2.65(0.76,4.53)	0.006	79	<0.00001
**Time to discharge, min**	5	475	WMD3.72(-2.80,10.24)	0.26	41	0.15
**PONV**	9	842	RR 2.23(1.33,3.77)	0.003	42	0.09

RR = relative risk; WMD = weighted mean difference; CI = confidence interval; EA = emergence agitation; PONV = postoperative nausea and vomiting; PACU = post anesthesia care unit.

We conducted subgroup analysis separately because confounding factors, such as premedication, type of surgery, preschool-aged children and pain, may have affected the incidence of EA (Table 3). Subgroup analysis of the timing of administration revealed that the use of fentanyl both before and at the end of surgery resulted in a preventive effect against EA (RR = 0.39, 95% CI 0.28~0.54, *P*<0.00001, *I* ^2^ = 59%; RR = 0.26, 95% CI 0.15~0.47, *P*<0.00001, *I* ^2^ = 0%). Analysis of 12 intravenous fentanyl trials showed that this intervention was effective (RR = 0.35, 95% CI 0.24~0.50, *P*<0.00001, *I* ^2^ = 53%). Three intranasal studies of this drug had RR of 0.30(95% CI 0.12~0.72, *P* = 0.008, *I* ^2^ = 50%), and one oral study had an RR of 0.54 (95%CI 0.35~0.83, *P* = 0.005). The effect of midazolam is still an ongoing debate. The meta-analysis by Zhang et al found midazolam had a significant effect on preventing EA [57] while the meta-analysis by Dahmiani et al found midazolam to be ineffective for the prevention of EA. On the contrary, midazolam might even trigger EA [5]. To eliminate the effects of this drug, we performed subgroup analysis, including 9 trials without premedication and 4 studies with midazolam premedication, and showed the prevention of EA in the fentanyl group (RR = 0.34, 95% CI 0.23~0.50, *P*<0.00001, *I* ^2^ = 55%; and RR = 0.34, 95% CI 0.13~0.90, *P* = 0.03, *I* ^2^ = 63%, respectively). Ear, nose and throat (ENT) procedures were reported to be independent risk factors for EA [58]. The protocols of 8 studies included ENT procedure for children. The pooled RR of ENT procedure studies was 0.45 (95% CI 0.32~0.64, *P*<0.0001, *I* ^2^ = 33%). When pooled analysis was limited to studies of patients who underwent minor urologic or inguinal surgery and received an appropriate regional block with enough local anesthetics, we found that the pooled RR was 0.34 (95% CI 0.21~0.57, *P*<0.0001, *I* ^2^ = 0%). Seven trials evaluated EA in preschool children younger than 7 years of age. We found that the pooled RR was 0.33 (95% CI 0.21~0.52, *P*<0.00001) but that the *I* ^2^ remained high at 59%.

**Table 3:** Effects of Subgroup Analysis on Meta-analysis Comparing fentanyl and placebo.

Subgroup	No. of studies	No. of participants	RR (95%CI)	*P*-value	*I* ^*2*^,%	Heterogeneity *P*-value
Timing of administration						
Before surgery	12	1074	0.39[0.28,0.54]	<0.00001	59	0.005
before the end of surgery	4	288	0.26[0.15,0.47]	<0.00001	0	0.99
Route of administration						
Intravenous	12	904	0.35[0.24,0.50]	<0.00001	53	0.01
Intranasal	3	333	0.30[0.12,0.72]	0.008	50	0.14
Oral	1	125	0.54[0.35,0.83]	0.005	NA	NA
Premedication						
without	9	766	0.34[0.23,0.50]	<0.00001	55	0.02
with midazolam	4	331	0.34[0.13,0.90]	0.03	63	0.05
Surgery						
ENT	8	681	0.45[0.32,0.64]	<0.0001	33	0.16
subumbilical	4	292	0.34[0.21,0.57]	<0.0001	0	0.61
Preschool children(aged<7 yr)	7	728	0.33[0.21,0.52]	<0.00001	59	0.02

NA = not applicable; OR = odds ratio; CI = confidence interval; Ear, nose and throat = ENT.

### Secondary outcomes

#### Pain incidence in PACU

**Fig 3:**
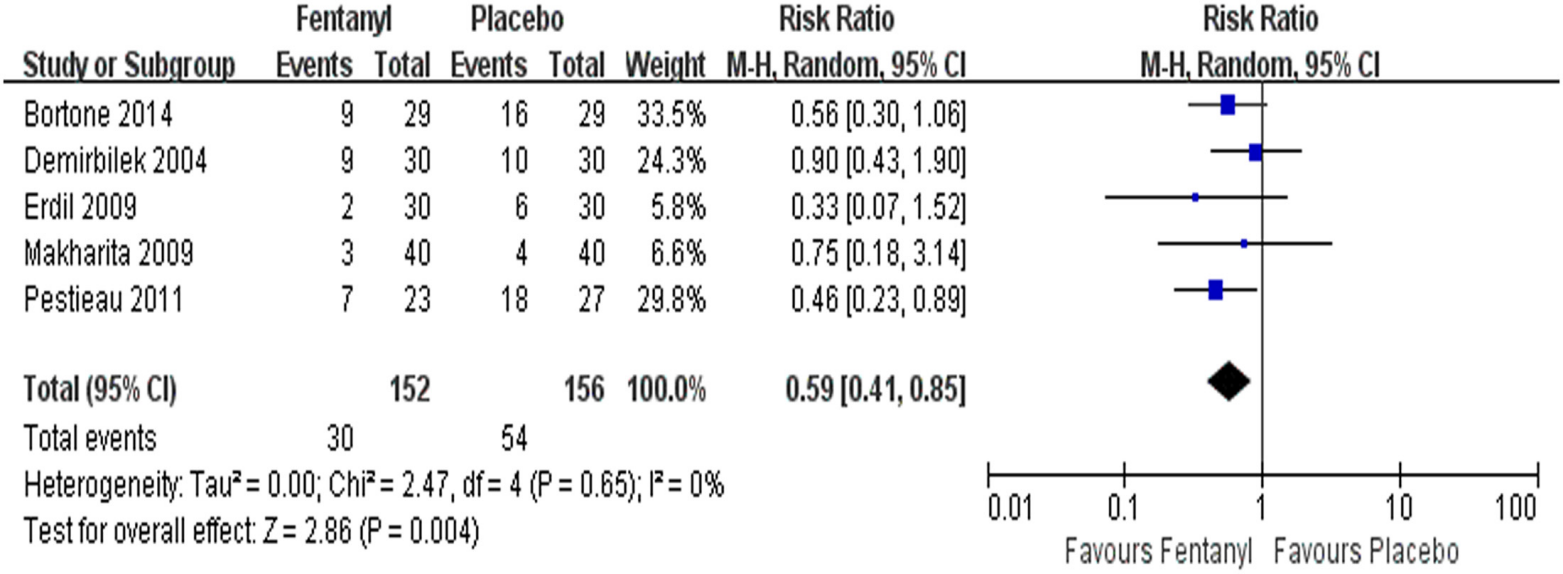
Forest plot and meta-analysis of pain incidence in PACU. EA = emergence agitation; M-H = Mantel-Haenszel method; CI = confidence interval; PACU = Postanesthesia care unit.

Five studies [4, 46, 50, 52, 56] (n = 308) were included in pooled analysis of pain incidence in the PACU between the fentanyl and placebo group. The data were homogeneous (*I* ^*2*^ = 0%, *P* = 0.65), and the pooled results suggested that fentanyl significantly decreased the incidence of pain in children in the PACU (RR = 0.59, 95%CI 0.41~0.85, *P* = 0.004) (Fig 3).

#### Extubation time

**Fig 4:**
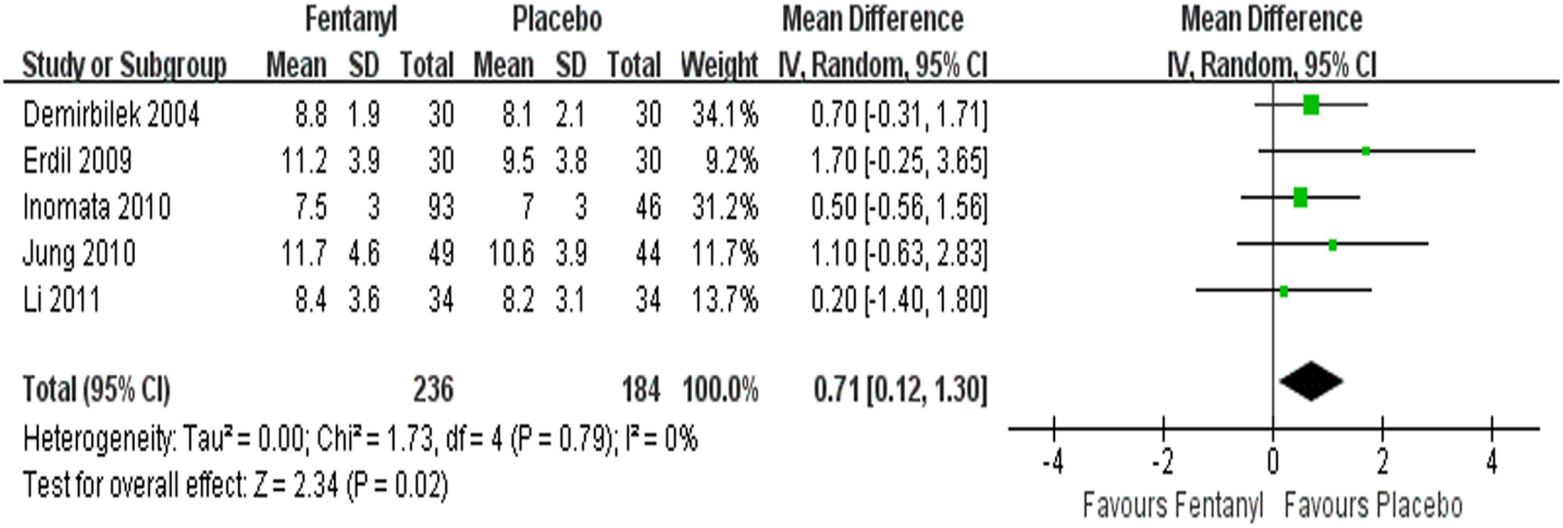
Forest plot and meta-analysis of extubation time. EA = emergence agitation; M-H = Mantel-Haenszel method; CI = confidence interval.

A total of 5 studies [45, 48–50, 52] reported extubation time in children with sevoflurane anesthesia, and the combined data suggested that it was prolonged by fentanyl (WMD = 0.71 min, 95% CI 0.12~1.30, *P* = 0.02). There was no heterogeneity among the results (*I* ^*2*^ = 0%, *P* = 0.79) (Fig 4).

#### Emergence time

**Fig 5:**
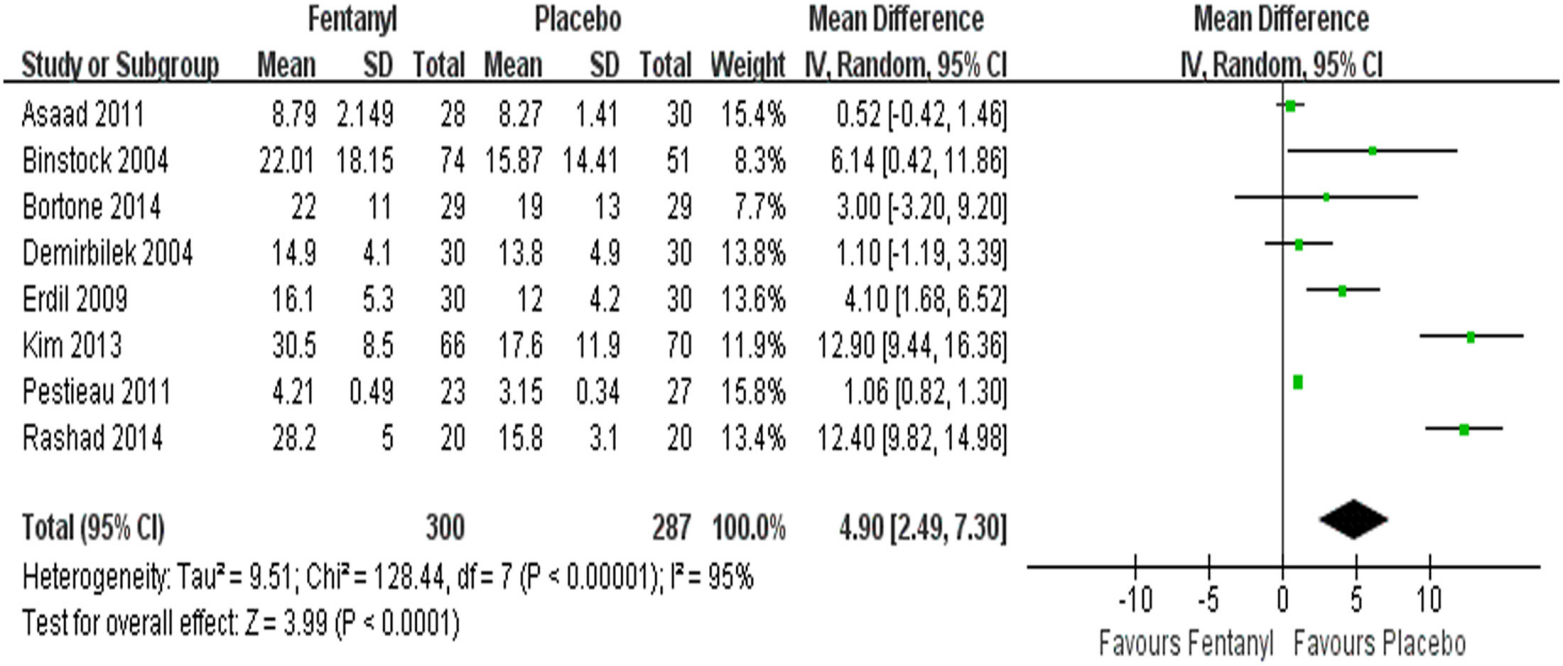
Forest plot and meta-analysis of emergence time. EA = emergence agitation; M-H = Mantel-Haenszel method; CI = confidence interval.

Emergence time was examined in eight studies [4, 43, 44, 46, 47, 50, 52, 53]. We found that the emergence time in the fentanyl group was longer than that in the control group (WMD = 4.90 min, 95%CI 2.49~7.30, *P*<0.0001) (Fig 5). The test for heterogeneity revealed an *I* ^*2*^ value of 95% (*P*<0.00001). When we removed the studies of Erdil 2009 [50], Kim 2013 [44], and Rashad 2014 [43], the heterogeneity was significantly decreased (*I* ^*2*^ = 14%, P = 0.33), and the pooled WMD was 1.04 min (95% CI 0.81~1.27, *P*<0.00001).

#### Time in PACU

**Fig 6:**
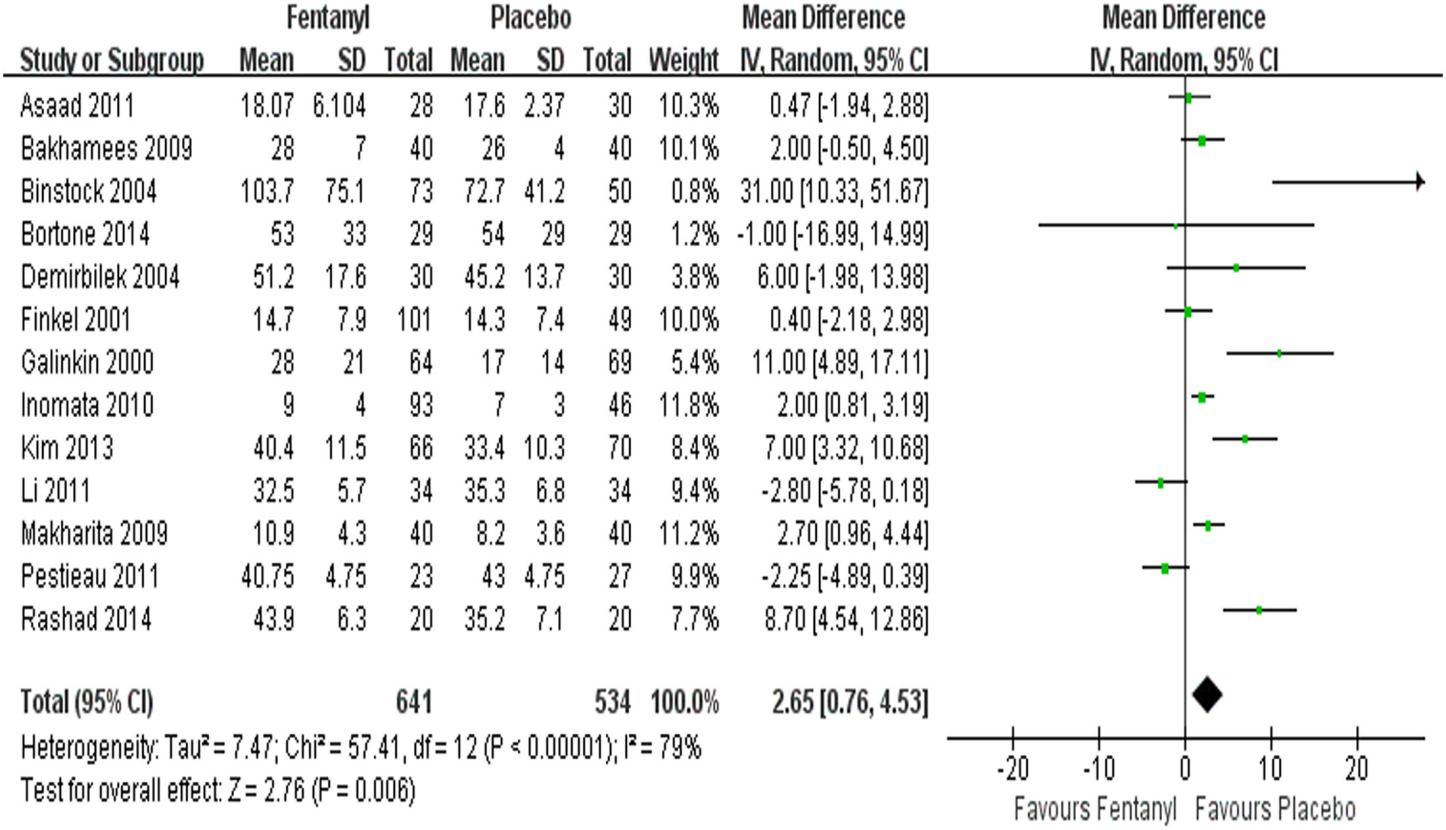
Forest plot and meta-analysis of time in PACU. EA = emergence agitation; M-H = Mantel-Haenszel method; CI = confidence interval; PACU = Postanesthesia care unit.

Time in the PACU was examined in 13 studies [4, 12, 43–48, 51–53, 55, 56]. The time in the PACU in the fentanyl group was longer than that in the control group (WMD = 2.65 min, 95% CI 0.76~4.53, *P* = 0.006). Because the *I* ^*2*^ value was 79% (*P*<0.00001), the random effects model was used to pool the data (Fig 6).

#### Time to discharge (day patients)

**Fig 7:**
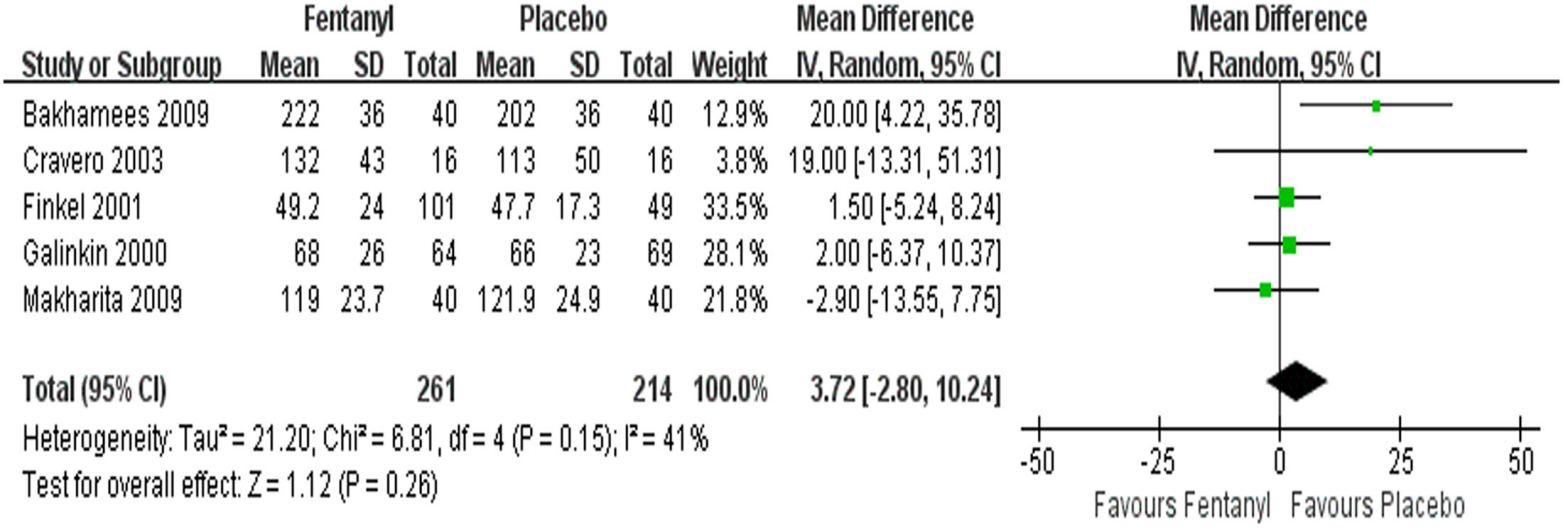
Forest plot and meta-analysis of time to discharge. EA = emergence agitation; M-H = Mantel-Haenszel method; CI = confidence interval.

The time to discharge of day patients was explored in 5 trials [12, 51, 54–56], and the data were homogeneous (*I* ^*2*^ = 41%, *P* = 0.15). The pooled data suggested that no evidence of a difference in time to discharge (WMD = 3.72 min, 95% CI -2.80~10.24, *P* = 0.26) between the fentanyl and placebo groups (Fig 7).

#### Adverse events

**Fig 8:**
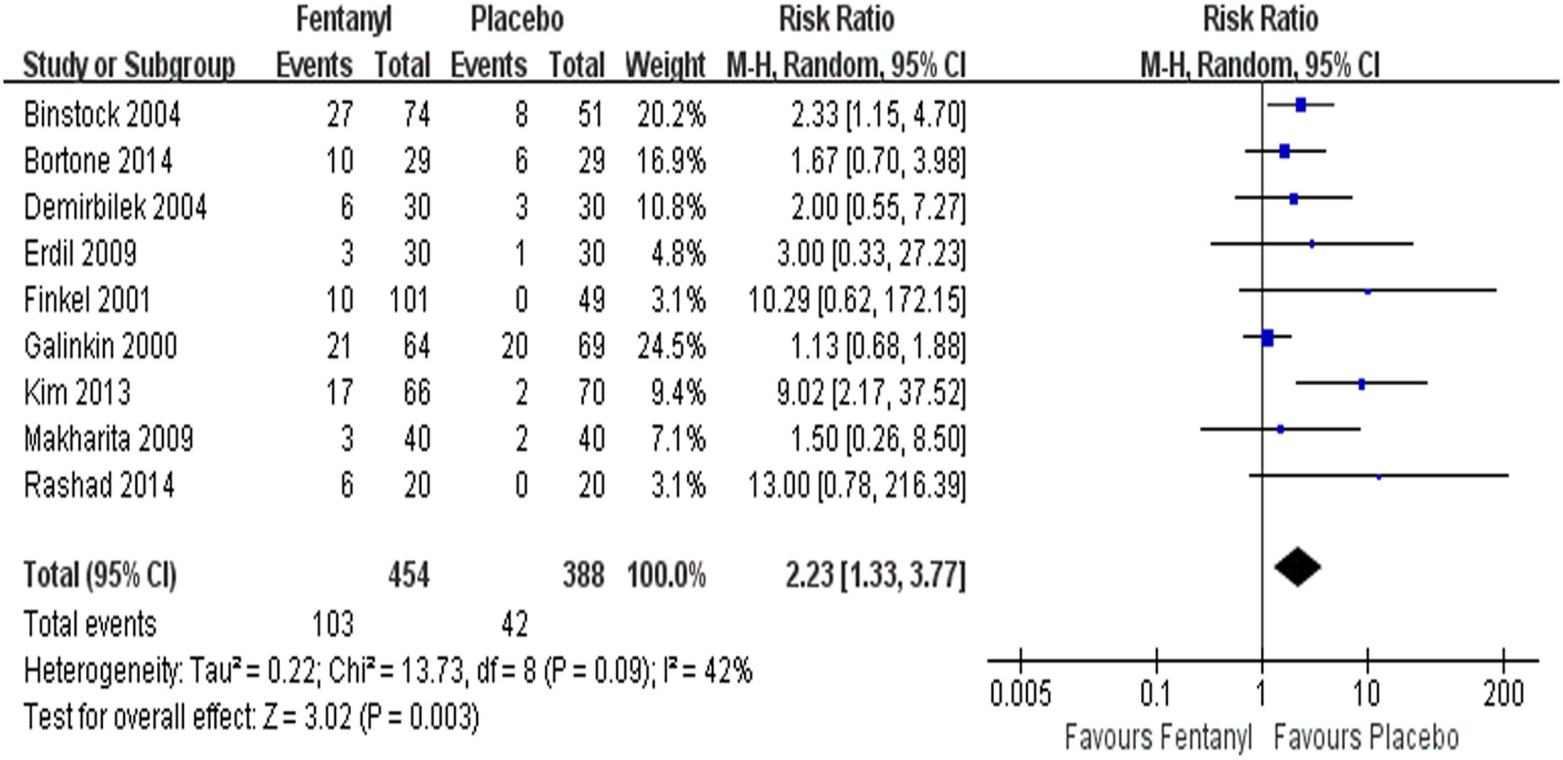
Forest plot and meta-analysis of PONV incidence. EA = emergence agitation; M-H = Mantel-Haenszel method; CI = confidence interval; PONV = postoperative nausea and vomiting.

The assessment of 9 studies [4, 12, 43, 44, 50, 52, 53, 55, 56]together showed that PONV occurred in 103 of 454 patients in the fentanyl group and 42 of 388 patients in the placebo group. The pooled results showed that fentanyl significantly increased the PONV incidence in the children under sevoflurane anesthesia (RR = 2.23, 95% CI 1.33~3.77, *P* = 0.003, *I* ^*2*^ = 42%) (Fig 8). One study [44] reported that a participant experienced suspicious laryngospasm, and 4(6%) patients had airway obstruction in the fentanyl group. Another study [53] showed that the risk of drug-related respiratory adverse events was higher for patients receiving oral transmucosal fentanyl citrate (OTFC) than for other patients; however, most of the adverse events were mild. No study reported hemodynamic events requiring intervention in any arm.

### Methodological qualities of included studies and potential sources of bias

The methodological qualities of the included trials were showed in Table 4. No study was found to beat a high risk of bias for any of the criteria considered. The blinding of participants and personnel, the blinding of the outcome assessment, the presence of incomplete outcome data, and selective reporting were determined to be at a low risk of bias in all included studies. Random sequence generation was unclear in five trials [45, 47, 49, 51, 53], and allocation concealment was unclear in 14 studies [4, 12, 43, 44, 46, 48–55].

**Table 4:** Risk of bias assessment for evaluation the quality of each included trials.

Author year	Random sequence generation	Allocation concealment	Blinding of participants and personnel	Blinding of outcome assessment	Incomplete outcome data	Selective reporting
**Bortone 2014**	low	unclear	low	low	low	low
**Rashad 2014**	low	unclear	low	low	low	low
**Kim 2013**	low	unclear	low	low	low	low
**Li 2011**	unclear	low	low	low	low	low
**Pestieau 2011**	low	unclear	low	low	low	low
**Asaad 2011**	unclear	low	low	low	low	low
**Inomata 2010**	low	unclear	low	low	low	low
**Jung 2010**	unclear	unclear	low	low	low	low
**Erdil 2009**	low	unclear	low	low	low	low
**Makharita 2009**	low	unclear	low	low	low	low
**Bakhamees 2009**	unclear	unclear	low	low	low	low
**Demirbilek 2004**	low	unclear	low	low	low	low
**Binstock 2004**	unclear	unclear	low	low	low	low
**Cravero 2003**	low	unclear	low	low	low	low
**Finkel 2001**	low	unclear	low	low	low	low
**Galinkin 2000**	low	unclear	low	low	low	low

**Fig 9:**
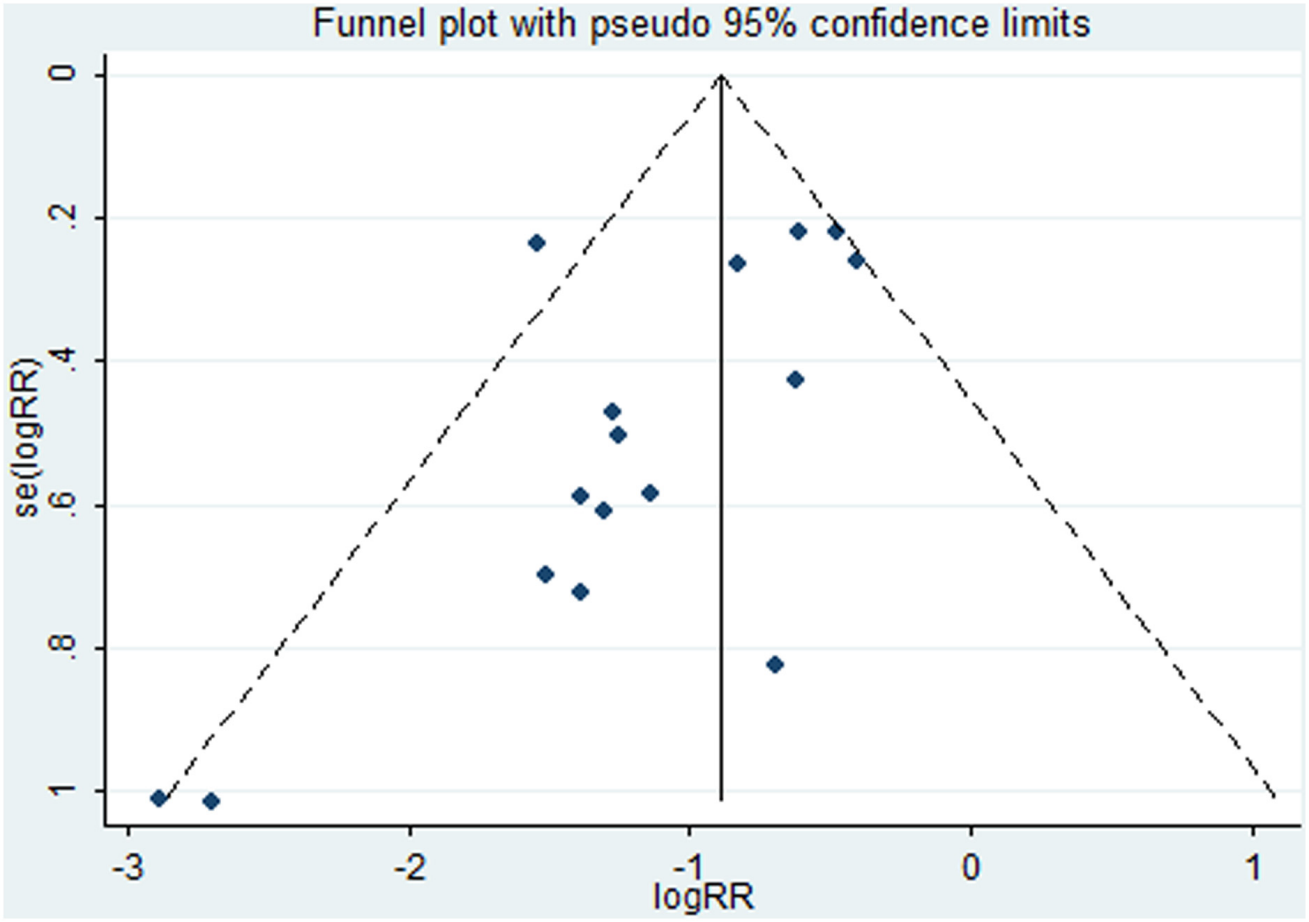
Funnel plots illustrating meta-analysis EA incidence. SE = standard error; RR = Relative risk; EA = emergence agitation.

A funnel plot of the included studies that reported the incidence of EA showed potential publication bias (Begg’s test, *P* = 0.022, Egger’s test, *P* = 0.023) (Fig 9). Considering the effect of the missing trials, we conducted a trim-and-fill analysis and the analysis showed “no trimming performed; data unchanged”.

## Discussion

This systematic review and meta-analysis of 16 RCTs, including 1362 patients, indicates that fentanyl significantly reduces the incidence of EA under sevoflurane anesthesia in children and decreases postoperative pain but it increases the incidence of PONV. The extubation time, emergence time, and time in the PACU were slightly prolonged. We found no significant difference in the time to discharge of day patients.

Several previous meta-analyses indicate that fentanyl can reduce the incidence of EA under sevoflurane anesthesia in children [59], whereas the meta-analysis by Dahmiani et al state that intravenous. fentanyl failed to prevent EA [5]. In our subgroup analysis, we found that both intravenous and intranasal fentanyl showed to be effective. The reasons for these conflicting results may be due to inclusion of only two studies in the meta-analysis by Dahmiani et al.

Fentanyl, a short-acting opioid analgesic, is used to reduce the incidence of pain. Some investigators have argued that pain experienced during impaired consciousness in children results in severe EA [13, 58, 60]. Our findings also showed that fentanyl decreased the incidence of pain in children in the PACU (RR = 0.59, 95%CI 95%CI 0.41~0.85, *P* = 0.004) and reduced the incidence of EA (RR = 0.37, 95% CI 0.27~0.49, *P*<0.00001) in children under sevoflurane anesthesia. However, it was still difficult to fully identify EA or pain-induced behavioral disorders in the children evaluated in the present study. Locatelli et al suggested that the splitting of PAED scale into ED1 and ED2 scores might help to separate ED from pain [61]. In addition, previous studies have reported a frequent incidence of EA in patients who have received sevoflurane for genitourinary surgery with an adequate caudal block and for non painful interventions, such as magnetic resonance imaging [7, 54]. Following restriction of the studies achieving a high level of pain relief during surgery by regional nerve block, the preventative effect of fentanyl remained significant (RR = 0.34, 95% CI 0.21~0.57, *P*<0.00001, *I* ^*2*^ = 0%). Thus, it is hard to establish an explicit relationship between pain and EA, and pain may not be the only factor affecting the occurrence of EA in children. Fentanyl is effective for EA in a rather unspecific way. Whatever the reason for EA might be pain, delirium, agitation for other reasons such as parental separation, hunger, thirst etc, fentanyl provides analgesia and sedation and hence disrupts agitation and crying. It resolves the problem even without knowing the exact underlying cause, especially in those situations where there might be an overlap between pain and delirium.

Some studies have demonstrated that rapid awakening is one of the factors contributing to EA [62] because of the low blood-gas solubility and rapid emergence characteristics of sevoflurane. In the current study, the children administered fentanyl were found to have a slightly prolonged extubation time (WMD = 0.71 min, 95% CI 0.12~1.3, *P* = 0.02, *I* ^*2*^ = 0%), emergence time (WMD = 4.90 min, 95% CI 2.49~7.30, *P*<0.0001, *I* ^*2*^ = 95%) and time in the PACU (WMD = 2.65 min, 95% CI 0.76~4.53, *P* = 0.006) and a lower incidence of EA. Some authors have found that the incidence of EA is not reduced by delayed emergence from sevoflurane anesthesia in children [63]. Therefore, it is still difficult to confirm that fentanyl reduces the incidence of EA by preventing rapid emergence from sevoflurane anesthesia.

The incidence of PONV was significantly higher in the fentanyl group than the placebo group (RR = 2.23, 95% CI 1.33~3.77, *P* = 0.003, *I* ^2^ = 42%). However, a lack of postoperative follow-up after more than 24 hours may have been a limiting factor in the interpretation of these study results. Other adverse events were reported in two studies; however, we did not find any serious adverse events in any of the included trials. Additional adverse events were infrequent in most studies mentioning ‘ no adverse events’ and in those not addressing them at all. Thus, we were notable to ascertain safety.

Between-study heterogeneity was significant for some of the continuous variables but was not significant for the dichotomous outcomes. Different surgery types, children’s ages, premedication, timing and the route of administration were described in the included studies. These differences may have resulted in the significant between-study heterogeneity. The effect of heterogeneity may have been reduced by using the random effects model, but not abolished.

Some limitations need to be considered for the present study. The main limitation is that the incidence of EA may have been greatly influenced by the uses different scales with different cut-off values to define the presence of EA and some of the scales are not validated [64]. Because small children cannot verbalize pain, anxiety, thirst or hunger, it is difficult to interpret their behaviors [65]. Although some studies used a reliable pain scale and the PAED scale to decrease errors associated with pain, a clear differentiation between EA and agitation because of pain could not be guaranteed. Future systematic reviews should explore different EA assessment tools separately when a sufficient amount of data is available. In addition, the follow-up time was generally short; therefore, any impacts on the long-term outcome of EA remain to be validated. Furthermore, we restricted the study selection to the English language and unpublished studies were not included in this meta-analysis adding a language bias and publication bias. Some studies reported that the exclusion of non-English studies may result in more conservative estimates of treatment effects, because studies with positive results were more likely to be published and more likely to be published in English [66]. Nevertheless, we searched for studies with multiple strategies, included and evaluated the methodological qualities of the studies with strict criteria, and minimized heterogeneity with subgroup analysis. Therefore, we provide the up-to-date information on this topic.

## Conclusions

In conclusion, this systematic review and meta-analysis indicates that fentanyl may be associated with a decreased incidence of EA in children under sevoflurane anesthesia in addition to reduced postoperative pain, but has a higher incidence of PONV. However, considering the inherent limitations of the included studies, more RCTs with extensive follow-up should be performed to validate our findings in the future.

## Supporting Information

S1 Checklist PRISMA Checklist. (DOC)

S1 File A list of full-text excluded articles. (DOCX)
