# Supplementary material for: Effects of Fentanyl on Emergence Agitation in Children under Sevoflurane Anesthesia: Meta-Analysis of Randomized Controlled Trials
Source: PLoS One. 2015 Aug 14;10(8):e0135244. doi: 10.1371/journal.pone.0135244 (PMC4537096; doi:10.1371/journal.pone.0135244)
Supplement: S1 File — (DOCX) [file pone.0135244.s002.docx]

**A list of full-text excluded articles with the reasons for exclusion**

**We excluded 24 full-text studies for the following reasons**

**Thirteen for the lack of a control group:**

[1] Li X, Zhang Y, Zhou M, et al. The effect of small dose sufentanil on emergence agitation in preschool children following sevoflurane anesthesia for elective repair of unilateral inguinal hernia[J]. Saudi Med J,2013,34(1):40-45.

[2] Hippard H K, Govindan K, Friedman E M, et al. Postoperative analgesic and behavioral effects of intranasal fentanyl, intravenous morphine, and intramuscular morphine in pediatric patients undergoing bilateral myringotomy and placement of ventilating tubes[J]. Anesth Analg,2012,115(2):356-363.

[3] Guan L, Wang R, Li Q. Clinical observation of applying sufentanil in pediatric neuroanesthesia[J]. The Journal of Clinical Anesthestology,2011,27(1004-5805(2011)27:7<644:SFTNYY>2.0.TX;2-H7):644-646.

[4] Rampersad S, Jimenez N, Bradford H, et al. Two-agent analgesia versus acetaminophen in children having bilateral myringotomies and tubes surgery[J]. Paediatr Anaesth,2010,20(11):1028-1035.

[5] Patel A, Davidson M, Tran M C, et al. Dexmedetomidine infusion for analgesia and prevention of emergence agitation in children with obstructive sleep apnea syndrome undergoing tonsillectomy and adenoidectomy[J]. Anesth Analg,2010,111(4):1004-1010.

[6] Patel A, Davidson M, Tran M C J, et al. Dexmedetomidine Infusion for Analgesia and Prevention of Emergence Agitation in Children with Obstructive Sleep Apnea Syndrome Undergoing Tonsillectomy and Adenoidectomy[J]. ANESTHESIA AND ANALGESIA,2010,111(4):1004-1010.

[7] Shen X, Li W. Pharmacodynamics of fentanyl for inhibition of emergence agitation after sevoflurane-remifentanil anesthesia in children[J]. Chinese Journal of Anesthesiology,2010,30(0254-1416(2010)30:3<303:FTNYZQ>2.0.TX;2-D3):303-305.

[8] M S M, Sm A. Oral midazolam with low dose ketamine, fentanyl, or ketoprofen for the prevention of emergence agitation after pediatric ambulatory surgery.[J]. Egyptian Journal of Anaesthesia,2008(1).

[9] Kim CH, Jo HY, Ham JY, et al. The Effect of Fentanyl and Remifentanil on the Side Effect after Sevoflurane Anesthesia in Children undergoing Herniorrhaphy[J]. Korean Journal of Anesthesiology,2007,53(5):609-614.

[10] Aouad M T, Kanazi G E, Siddik-Sayyid S M, et al. Preoperative caudal block prevents emergence agitation in children following sevoflurane anesthesia[J]. Acta Anaesthesiol Scand,2005,49(3):300-304.

[11] Cohen I T, Finkel J C, Hannallah R S, et al. The effect of fentanyl on the emergence characteristics after desflurane or sevoflurane anesthesia in children[J]. Anesth Analg,2002,94(5):1178-1181.

[12] Liang P, Zhou C, Ni J, et al. Single-dose sufentanil or fentanyl reduces agitation after sevoflurane anesthesia in children undergoing ophthalmology surgery[J]. Pak J Med Sci,2014,30(5):1059-1063.

[13] Abdelhalim A A, Alarfaj A M. The effect of ketamine versus fentanyl on the incidence of emergence agitation after sevoflurane anesthesia in pediatric patients undergoing tonsillectomy with or without adenoidectomy[J]. Saudi J Anaesth,2013,7(4):392-398.

**One for being older than 14 years:**

[1] Hung W T, Chen C C, Liou C M, et al. The effects of low-dose fentanyl on emergence agitation and quality of life in patients with moderate developmental disabilities[J]. J Clin Anesth,2005,17(7):494-498.

**Three for the lack of availability of a full-text version:**

[1] Finkel J, Cohen I T, Kim M, et al. Effect of Intranasal Fentanyl on Emergence Following Sevoflurane Anesthesia for BMT Surgery in Children.[J]. Anesthesiology Abstracts of Scientific Papers Annual Meeting,2002(2000):1252.

[2] Galinkin J L, Fazi L M, Cuy R M, et al. Intranasal Fentanyl in Children Undergoing Bilateral Myringotomy and Tube Placement.[J]. Anesthesiology Abstracts of Scientific Papers Annual Meeting,2002(2000):1253.

[3] Cravero J P, Thyr B, Beach M, et al. The Effect of Intravenous Fentanyl Agitation in Pediatric Patients.[J]. Anesthesiology Abstracts of Scientific Papers Annual Meeting,2002(2001):1222.

**Seven for not being written in English:**

[1] Zhang Y, Liu J, Wu X, et al. Effect of dezocine on emergence agitation during recovery from sevoflurane-based anesthesia in children[J]. Chinese Journal of Anesthesiology,2012,32(0254-1416(2012)32:12<1425:DZXDHE>2.0.TX;2-412):1425-1428.

[2] Pei C, Li T. Different drugs on preventing sevoflurane-induced post-anesthesia agitation in children[J]. The Journal of Clinical Anesthestology,2012,28(1004-5805(2012)28:1<17:BTYWYF>2.0.TX;2-31):17-18.

[3] Kim HG, Ban JS, Lee JM, et al. The Effect of Alfentanil on the Emergence Agitation after Sevoflurane Anesthesia in Children Undergoing Inguinal Herniorraphy[J]. Korean Journal of Anesthesiology,2005,49(3):370-375.

[4] Park SY, Kim JY, Gwak HJ, et al. The Effect of Ketamine and Fentanyl on the Incidence of Emergence Agitation after Sevoflurane Anesthesia in Children undergoing Tonsillectomy[J]. Korean Journal of Anesthesiology,2005,49(4):502-506.

[5] B I, M A, At D, et al. The effect of fentanyl administration at different periods on emergence agitation. [Turkish][J]. Turk Anesteziyoloji ve Reanimasyon Dernegi Dergisi,2005(5).

[6] Jin H C, Lee SJ, Seok L J, et al. The Effect of Fentayl and Midazolam on the Incidence of Emergence Agitation in ChildrenFollowing Sevoflurane Anesthesia for Tonsillectomy[J]. Korean Journal of Anesthesiology,2004,46(5):524-527.

[7] Mj I, Dy K, Ch K, et al. The effect of ketorolac and fentanyl on the emergence characteristics after sevoflurane anesthesia in children undergoing tonsillectomy and adenoidectomy [Korean][J]. Korean journal of anesthesiology,2004(1).
